# Follow-up Study Methods for a Longitudinal Cohort of Alaska Native and American Indian People Living within Urban South Central Alaska: The EARTH Study

**DOI:** 10.1007/s10900-019-00630-z

**Published:** 2019-02-23

**Authors:** Julie A. Beans, Vanessa Y. Hiratsuka, Aliassa L. Shane, Gretchen E. Day, Diana G. Redwood, Christie A. Flanagan, Amy Swango Wilson, Barbara V. Howard, Jason G. Umans, Kathryn R. Koller

**Affiliations:** 1 Research Department, Southcentral Foundation, 4085 Tudor Centre Drive, Anchorage, AK 99508 USA; 2 Alaska Native Tribal Health Consortium, 3900 Ambassador Dr., Ste. 201, Anchorage, AK 99508 USA; 3 Georgetown-Howard Universities Center for Clinical and Translational Science, Washington, DC USA; 4 MedStar Health Research Institute, Hyattsville, MD USA

**Keywords:** Cohort studies, Methods, Alaska Native, American Indian, Longitudinal studies, Measurement

## Abstract

Longitudinal data are needed to investigate chronic disease causation and improve prevention efforts for Alaska Native and American Indian (ANAI) people. This paper describes the methods used to conduct follow-up data collection of a longitudinal cohort that enrolled ANAI adults between 2004 and 2006 in south central Alaska. The follow-up study re-examined ANAI participants in a large, urban centre in south central Alaska between 2015 and 2017. Computerized surveys were used to collect self-reported health, lifestyle, physical activity, and diet data. Clinical measurements included blood pressure, fasting blood glucose and lipid panel, urine albumin/creatinine, height, weight, and waist and hip circumference. Participants were provided individual results at the conclusion of their visit. A total of 1320 south central Alaska study participants completed the baseline visit. Study staff attempted to contact all living cohort members for inclusion in the follow-up study. More than 11,000 attempted contacts were made. Of the 637 available for participation, 388 completed the follow-up visit. The proportion of women increased from baseline to follow-up examinations (67 vs. 72%, p < 0.01). Self-reported health status of being married or living as married (46% vs. 39%, p < 0.01), and those reporting being employed or self-employed (55% vs. 47%, p < 0.01) were higher at follow-up when compared to baseline. Almost all participants at follow-up (97%) agreed to long-term storage of biological specimens for future study. Despite demographic differences between the follow-up and baseline cohorts, longitudinal data collected will provide novel insight on chronic disease development and prevention for ANAI people as well as other populations.

## Introduction

Improvements in public health—such as vaccination programs, water/sanitation projects, and the provision of community-based primary and emergency health care with access to secondary and tertiary health care—within the Alaska Tribal Health System has resulted in increased lifespans since the mid-1950s for Alaska Native and American Indian (ANAI) people living in Alaska [1]. Increased life expectancy has shifted health care focus from communicable to chronic diseases. This shift in focus has led several ANAI communities to redefine their health and health care priorities on the basis of research findings.

One example is the Alaska Education and Research Towards Health (EARTH) study, conceived originally as one of three linked ANAI cohort studies, that was designed to investigate the role of diet, physical activity, and other lifestyle and cultural factors in the development of chronic disease among ANAI people [2]. The initial project was coordinated by researchers based at the University of Utah who partnered with tribal health organizations, including the Alaska Native Tribal Health Consortium (ANTHC) and Southcentral Foundation (SCF), both tribal health organizations serving ANAI people in Alaska.

Baseline Alaska EARTH data identified important cross-sectional associations between disease prevalence and behavioral, cultural, and environmental factors [2–26]. Longitudinal data are, however, crucial to elucidating disease causation and outcomes, and designing community-specific prevention efforts. The Alaska EARTH Follow-up study (“follow-up study” hereafter) was designed by Alaska Native tribal health organization-based researchers to re-examine the SCF subset of Alaska EARTH cohort participants enrolled 10–12 years prior. We describe the methods employed in this follow-up study using the baseline study measurement tools while adapting to the many behavioral, social, environmental, technological, and other changes that occurred during the intervening decade. We describe similarities and differences in baseline characteristics of the original and follow-up cohorts that require consideration when generalizing follow-up results to the original SCF cohort or to ANAI living in the urban Anchorage area of Alaska.

## Methods

The follow-up study was led by ANTHC, which partnered with SCF and MedStar Health Research Institute (MHRI) to complete a second examination among Alaska EARTH participants in southcentral Alaska focused on incident diabetes and associations with baseline risk and protective factors. A secondary aim was to assess the incidence of metabolic syndrome components and their associations with baseline characteristics, diet, physical activity, and subsistence lifestyle.

### Participants and Recruitment

Of 3821 total Alaska EARTH participants enrolled in the baseline study between 2004 and 2006 in three regions of Alaska [2], 1320 participants were enrolled at the SCF recruitment site. Eligibility requirements for the follow-up study included participation in the baseline EARTH study conducted at SCF and the ability to read and understand a consent form, complete survey instruments and medical tests, and complete the interview in English. Deferred enrollment was offered to participants who were pregnant (until 3 months post-partum) or actively undergoing cancer treatment (until one year following cancer treatment completion).

Research staff modified the original EARTH Study Microsoft Access® database to document and track participant re-contact attempts. The database consisted of contact information for each participant from the recruitment site’s baseline EARTH cohort. With SCF and ANTHC privacy officer permission, contact information (telephone number, email address and mailing address) was updated for each participant from the ANTHC and SCF co-managed electronic medical record (EMR). Deceased participants, identified using state vital statistics records, were excluded from recruitment efforts. The database included the number and outcomes of participant contacts.

Follow-up study recruitment took place between June 2015 and September 2017, the majority by telephone contact. Recruitment and data collection staff used several strategies to provide baseline EARTH participants the opportunity to participate in the follow-up study. Key outreach strategies included using multiple contact methods: telephone contact, postal and electronic mail, and in-person encounters; employing members of the ANAI community as study staff members; conducting data collection at local ANAI community health locations; providing incentives for participation (including real-time return of results in the form of an health risk assessment debrief at the conclusion of the visit); and using a database containing all contact history and contact information to effectively engage study participants.

### Study Staff Training

The follow-up study team consisted of AN and/or AI tribal members employed by SCF in pre-baccalaureate to post-doctoral positions. Each follow-up study team member completed about 40 h of EARTH study-specific training in addition to IRB-required trainings for the protection of research participants prior to conducting study visits (Table 1). Study-specific trainings included confidentiality training, Cholestech® portable lab analyzer certification, certification in EARTH-standardized clinical/anthropometric data collection procedures, biohazard and laboratory safety training, biospecimen tracking and processing procedures, biospecimen freezer use and maintenance procedures, and review and update of the study manual of operations. Training also included appointment scheduling and tracking, informed consent procedures, interviewer-administered demographic data intake, participant self-administered questionnaires, follow-up data collection via exit interview, and participant feedback.

**Table 1:** EARTH study staff training log

Study staff name	
Confidentiality	
Confidentiality agreement	SCF employee annual orientation
Collaborative Institutional Training Initiative (CITI)—human research	
Medical test/measurement certification	
Cholestech® portable analyzer	Waist and Hip circumference
Weight	Blood pressure
Height	Biohazard training
Laboratory training	
Lab safety	Sample tracking
Sample processing	Freezer storage
Study component certification	
Manual of operations review	Self-administered questionnaires
Scheduling procedures	
Participant tracking	Exit interview
Informed consent process	Feedback
Intake interview/data entry	Data back-up procedures

### Follow-up Study Visit

As with the baseline study setting at SCF, the majority of follow-up visits were conducted in a clinic room at the SCF Anchorage Native Primary Care Center (ANPCC) during regular business hours. Follow-up visits also took place in clinic exam rooms at the SCF Valley Native Primary Care Center (VNPCC) located in Wasilla, Alaska, which is within the urban Anchorage vicinity. The follow-up study visit mirrored the original baseline visit (Table 2), consisting of informed consent; intake questionnaire; anthropometric, lipid, and glucose measurements, and multiple computerized self-interview questionnaires [2]. In addition, the follow-up study included blood and urine sample collection, and participants were offered long-term blood sample storage in the Alaska Area Specimen Bank for future research use. Follow-up study participants received a $30 gift card.

**Table 2:** Baseline and follow-up study visit components

Measure	Instrument/source	Data collection follow-up only or both
Fasting insulin	Venipuncture	Follow-up
Glycated hemoglobin (HbA1c)	Venipuncture	Follow-up
Urine albumin/creatinine	Urine sample	Follow-up
Fasting lipids	Cholestech LDX®	Both
Fasting glucose	Cholestech LDX®	Both
Diet history questionnaire	Adapted from coronary artery risk development in young adults (CARDIA) study diet history questionnaire	Both
Nutrient content	Nutrition data system for research software	Both
Health, lifestyle, and physical activity questionnaire	Adapted from multi-ethnic study of atherosclerosis (MESA)	Both
	Adapted from Taylor physical activity questionnaires	
Demographic and medical history questionnaire	EARTH questionnaire	Both
Diagnosed type II diabetes cases	Medical records abstraction	Both
Blood pressure	Omron intelliSense blood pressure monitor (Hem-907/907XL)	Both
Body mass index	Tanita digital scale and road rod stadiometer	Both
Waist circumference	Novel products figure finder tape	Both

### Interview Data Collection

The follow-up study visits completed between June 2015 and August 2017 at ANPCC used the Study Computer Assisted Participant Evaluation System (SCAPES) developed for the baseline study [2, 25]. Additionally, the Research Electronic Data Capture (REDCap) system was used to provide a more portable data collection system than the original baseline study system. Data collection in the ANPCC location used a combination of SCAPES and REDCap, while the VNPCC location used REDCap only.

The EARTH intake; diet; health, lifestyle and physical activities; Alaska-specific; and exit interview questionnaires were recreated in REDCap by ANTHC study staff. The intention was to guide a follow-up study participant through the multiple EARTH interviewer- and self-administered questionnaires in a manner similar to SCAPES. Additionally, a participant feedback print-out with the re-exam Cholestech® blood test results and clinical measurements was designed to also mimic SCAPES.

### Medical Measurements

As in the baseline study, medical measurements included blood pressure, weight, height, waist and hip circumference, and blood lipid and glucose levels [2]. Measurement instruments and protocols for the blood pressure, weight, height, waist and hip circumference measurements used in the baseline EARTH study [2] were also used in the follow-up study.

Participants were asked to fast for 8 h prior to the study visit. Those who did not fast were given the option to reschedule the entire appointment or complete the visit except for the blood draw and reschedule an appointment to complete the fasting blood draw. Participants could take their usual medications with water during the 8-h fast. In the baseline study, blood samples for point-of-care (POC) testing of fasting lipids and glucose levels were collected by finger stick method and tested using the portable Cholestech LDX® analyzer system [2]. Unique to the follow-up study, investigators offered participants the opportunity to provide venipuncture samples. The follow-up study team worked with the clinics to provide participants a study experience with familiar staff and environment. A certified medical assistant clinic staff member performed the venipuncture and collected urine samples. Study staff used small portion of the venipuncture sample for the Cholestech LDX ® to provide POC lipid and glucose results as a component of immediate participant feedback. The remaining blood and urine specimens were processed by follow-up study staff, aliquoted, and stored at − 80 °C for later analyses. In addition, serum and plasma aliquots were deposited with the Alaska Area Specimen Bank and stored at − 30 °C for future research per participant consent [27].

### Participant Feedback

As with the baseline study visit, a participant feedback report was generated after completion of the follow-up questionnaires [2]. Participants were also given a copy of their medical measurements from baseline for comparison. Study staff reviewed baseline and follow-up study fasting glucose and lipid values as well as anthropometric measures with participants.

### Medical Record Abstraction

As in the baseline study, participant consent allowed a research nurse to review all follow-up participant EMRs from baseline to study date using the baseline study protocol. The EMR review included abstraction of all International Classification of Diseases, Ninth Edition (IDC-9) codes listed in the original EARTH abstraction protocol [9]. Additional data collected in the follow-up EMR review included presence of the diagnosis code for osteoporosis and prescriptions for several antihypertensive and lipid-lowering medications. Additional data items not included in the baseline EMR review were entered in the follow-up database with the initial date of entry in the EMR so that ICD-9 codes present at baseline could be distinguished from diagnostic codes appearing since study enrollment. All medical record data were anonymized and entered in a follow-up study Microsoft Access® database.

### Statistical Analysis

We compared baseline demographic characteristics of the overall original SCF cohort with the SCF follow-up cohort. The follow-up cohort total consisted only of those participants who completed their follow-up exam with anthropometric measurements, blood glucose and lipid testing, and intake and self-administered questionnaires. Baseline differences between the two groups were evaluated using Chi square tests for categorical variables and t-tests for linear variables using SAS Version 9.4 software (SAS Corporation Cary, NC, USA). P < 0.05 was considered statistically significant.

## Results

**Fig. 1:**
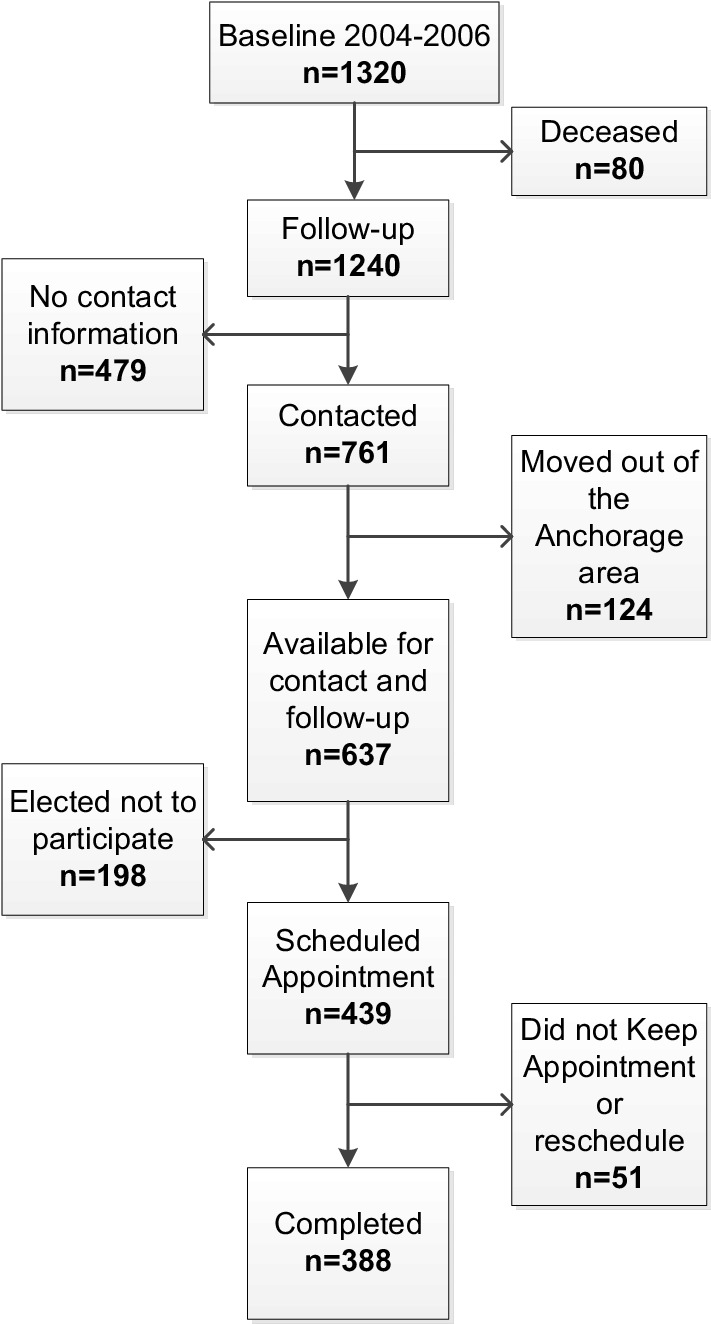
Flow chart outlining formation of the follow-up EARTH subset from the baseline cohort

While 1320 participants from the south central Alaska region initially enrolled at baseline, multiple factors reduced the cohort size substantially over the intervening decade (Fig. 1). A total of 80 (6%) participants were deceased by time of follow-up, leaving 1240 potential participants. A large proportion of participants did not have valid contact information (n = 479, 39%). Another 124 (10%) participants contacted had moved away from the study catchment area and elected not to participate in the follow-up exam. A total of 637 (51%) participants remained available for contact and follow-up.

Study staff attempted to contact all living cohort members for inclusion in the follow-up study. More than 11,000 attempted contacts were made, an average of nine contact attempts per participant completing a follow-up exam. Of the 637 available for participation, 198 (31%) individuals declined. Reasons included: lack of transportation, caregiving for children and/or Elders, incarceration, unable to leave work for study visit, not wanting to have venipuncture, and being ill or recovering from illness. Staff scheduled 898 appointments among 439 participants, as some participants scheduled multiple visits to complete the study. Of these, 208 appointments were not kept; and 212 appointments were rescheduled. In total, 388 (61% of the 637 total available participants) completed the follow-up study visit.

Comparing demographic characteristics of follow-up and baseline cohorts, most baseline characteristics were similar in the two groups, but proportions were exaggerated in the follow-up cohort, including the proportion of female participants, educational attainment, marital status, current employment, household income, language spoken at home, and self-reported health status (Table 3). There were no statistically significant differences in baseline age distribution or household size between the larger original cohort and the smaller follow-up subset.

**Table 3:** Comparison of baseline characteristics of the original south central Alaska EARTH study cohort and the follow-up subset

	Baseline cohort n (%)	Follow-up cohort n (%)	p-value^a^
All participants	1320 (100)	n = 388 (29.4)	
Sex			
Female	878 (66.5)	280 (72.2)	0.005
Age (years)			
18–34	486 (36.8)	129 (33.3)	0.217
35–54	683 (51.7)	211 (54.4)	
55+	151 (11.4)	48 (12.4)	
Household size^b^	3.5 (2.2)	3.4 (2.1)	0.225
Education level			
Greater than high school	712 (53.9)	253 (65.2)	< 0.0001
Marital status			
Married/living as married	511 (38.7)	178 (45.9)	0.0006
Employment status			
Currently employed or self-employed	618 (46.8)	213 (54.9)	0.0001
Income			
> $15,000^c^	758 (64.6)	273 (74.8)	< 0.0001
Length of time in residence (years)			
> 5^d^	514 (39.6)	150 (39.3)	0.86
Language spoken at home			
English^e^	1058 (80.3)	332 (85.8)	0.009
Self-reported health status			
Excellent/very good/good^f^	1015 (77.0)	322 (83.2)	0.0005
Family history of heart disease^g^	425 (55.8)	150 (58.4)	0.304
Family history of diabetes^h^	442 (57.5)	148 (57.8)	0.894

^a^Chi-square p-value for differences in proportions between respondents followed-up compared to those lost to follow-up

^b^Missing household size values for 10 baseline and 1 follow-up participants

^c^Missing income values for 147 baseline and 23 follow-up participants

^d^Missing residence values for 23 baseline and 6 follow-up participants

^e^Missing language value for 2 baseline and 1 follow-up participants

^f^Missing health status value for 1 baseline and 1 follow-up participant

^g^Missing family history of heart disease value for 558 baseline and 131 follow-up participants

^h^Missing family history of diabetes value for 637 baseline and 132 follow-up participants

Participants were given the option to have a portion of their biological specimens retained in long-term storage at the Alaska Area Specimen Bank [27] for future research on chronic disease. Approximately 97% of the follow-up cohort agreed to long-term biological specimen storage for future research.

## Discussion

This 10- to 12-year follow-up of a subset of the original EARTH cohort in the Anchorage area is one of very few to collect data to assess longitudinal changes in chronic disease risk and protective factors among ANAI populations [28–30]. Original study methods were carefully integrated into all aspects of the follow-up study; however, it was necessary to update some study instruments and data collection procedures.

The greatest limitation of this study was the small size of the final follow-up cohort relative to baseline, largely due to lack of current contact information. There was recognition at baseline that regular contact with participants was essential to ensure connection and retention [13, 16]. However, due to an extended funding gap from 2008 to 2014, no staff or resources were available to maintain or update contact information.

Recruitment strategies between the baseline and follow-up studies necessarily differed, with a lobby-based, convenience sample at baseline and primarily telephonic outreach to previously enrolled participants at follow-up. The voluntary provision of multiple contact telephone numbers by participants at the baseline exam proved inadequate without interim updates. Likewise, while contact information for all patients receiving care at the SCF clinics is updated in the EMR at each clinic visit, this proved inadequate as well, due to either infrequent medical care encounters or outdated/invalid information. Although updates from the EMR were useful in some instances, 479 of 1240 (39%) living baseline participants did not have valid contact information during follow-up study contact attempts. In the future, multiple contact numbers and other forms of continuous or automated electronic engagement, such as e-mail or social media contacts might better support recruitment and ongoing cohort engagement.

Within the follow-up study, successful recruitment methods included: reaching out to cohort members by multiple contact methods: mail, telephone, or in-person; employing well-trained study staff who were themselves members of the ANAI community; collecting data at an ANAI community health location; providing incentives for participation, including real-time return of health assessment results; and maintaining a database to record all participant contact history and contact information. Ineffective recruitment methods included weekend study visits. At baseline, participants were asked to provide up to three alternate contacts study staff could outreach to for updated contact information in the event the participant was unable to be reached. These alternate contacts yielded few updates to participant contact information. Alternate contact information was frequently outdated and when the alternate contact could be reached, the contact often stated that they were no longer in contact with the participant. Among those in contact with the participant, alternate contacts were often unwilling to relay messages or share participant contact information with the study team.

Adequately powered observational studies to assess chronic disease outcomes over the lifespan depend on long-term follow-up of large cohorts. Large cohort sizes are particularly challenging in small populations, such as ANAI people living in Alaska. In Alaska, difficulty in retention is compounded by geographic isolation in rural communities and high mobility of ANAI people within and between regions of the state and beyond the state due to limited resources within Alaska [31]. Although the original EARTH study team used custom software and then state-of-the-art touchscreen technology for baseline data collection, those products are now outdated. Recent advances in small, portable devices, and web-based software such as REDCap (as used in the current study), should enhance retention of mobile ANAI cohort members in the future.

The majority of follow-up participants (97%) agreed to biobanking of their samples in long-term storage at the Alaska Area Specimen Bank for use in future chronic disease research. This surprisingly high proportion of participation in biobanking may have been facilitated by the co-management of the Alaska Area Specimen Bank by ANAI organizations [32]. The follow-up study informed consent process for biobanking followed previously published recommendations for an informed consent process that clearly specified the data and/or biological specimens collected; the study questions; and specifics about specimen storage, destruction, and possible ongoing use [32].

The interactive model of the EARTH study with data input during the visit, same day clinical measurement feedback, and assistance with interpretation of results was a well-received method for gathering data while providing immediate feedback on current exam results. Participants had the opportunity to ask questions about their health and compare their health information from 10 or more years ago with current health information. Information for various SCF health education programming, such as exercise class schedules and tobacco cessation programming, were also made available to study participants.

Since 2000, ANTHC and SCF have increasingly engaged in research partnerships with academic institutions [21, 29, 33–41]. These research partnerships, including the original EARTH study, strengthened ANAI organizational research capacity by providing experience to apply for funding and carry out successful research projects for the tribal communities they serve. Between 2013 and 2018, ANTHC and SCF managed all facets of the follow-up study. ANTHC study staff trained during the baseline EARTH study provided the extensive training required to conduct this follow-up project. SCF research department ANAI staff were responsible for the follow-up visit effort. The ANAI staff members learned the skills necessary to carry out complex data collection for health research. Follow-up study staff members provided regular input on study processes, a practice aligned with the SCF corporate objective of shared responsibility [42]. Finally, the constant presence of follow-up study staff in the clinical setting was integral for both community members and SCF clinic staff to become familiar with the research process. This familiarity will contribute to better collaboration on health research projects in the future.

## Conclusion

Next steps in the follow-up study include analysis of the longitudinal data to investigate predictors of chronic disease, specifically diabetes and metabolic syndrome. Investigators are interested in both risk and protective factors in the biomedical, socio-behavioral, cultural, and environmental data collected. Dissemination of research findings to the ANAI and broader scientific communities will be a vital part of these next steps. Pending additional funding, the study team will plan for additional rounds of data collection to fulfill the original aim of the cohort: to increase knowledge of the unique protective and risk factors influencing health among ANAI people.

Despite limited retention, this is one of the larger and longest longitudinal studies of this understudied ANAI population focused on changes in cardiometabolic risk and protective factors to address chronic disease development and support prevention efforts. Lessons learned should inform the design of other longitudinal studies in this and similar populations.
